# Exploration of Perceived Determinants of Disordered Eating Behaviors in People with Mental Illness—A Qualitative Study

**DOI:** 10.3390/ijerph20010442

**Published:** 2022-12-27

**Authors:** Annabel S. Mueller-Stierlin, Anna Peisser, Sebastian Cornet, Selina Jaeckle, Jutta Lehle, Sabrina Moerkl, Scott B. Teasdale

**Affiliations:** 1 Department of General Practice and Primary Care, Ulm University Hospital, 89070 Ulm, Germany; 2 Department of Psychiatry and Psychotherapy II, Ulm University, 89070 Ulm, Germany; 3 Department of Psychiatry and Psychotherapeutic Medicine, Medical University of Graz, 8036 Graz, Austria; 4 Discipline of Psychiatry and Mental Health, University of New South Wales, Sydney, NSW 2052, Australia; 5 Mindgardens Neuroscience Network, Sydney, NSW 2052, Australia

**Keywords:** mental illness, depression, psychosis, bipolar, diet, nutrition, qualitative study, determinants

## Abstract

Disordered eating behaviors are common in people with a serious mental illness (SMI) such as schizophrenia, bipolar disorder and major depressive disorder. This study employed qualitative exploration to understand the perceived determinants of eating behaviors, in particular those connected to disordered eating patterns, in people with SMI. In total, 28 semi-structured interviews were conducted in a consecutive sample of people with SMI under treatment in local mental health services in Australia (*n* = 12), Germany (*n* = 8) and Austria (*n* = 8) (mean age: 43.3 years, proportion of female participants: 61%, proportion of participants with ICD-10 F2 diagnosis: 57%, proportion of participants with ICD-10 F3 diagnosis: 64%). A thematic analysis approach, the framework method, was applied using MAXQDA 2020. Three main themes of determinants were derived: (i) impacts to daily functioning, (ii) disrupted physical hunger cues and (iii) emotional hunger. For impacts to daily functioning, the following themes emerged: lack of daily structure, time and drive, and difficulty planning ahead. For physical hunger, themes emerged for disrupted hunger and satiety cues, and mindless eating. All motives listed in the Palatable Eating Motives Scale (PEMS), i.e., coping, reward, social and conformity, have been reported by participants to be drivers for their emotional eating behavior. Subsequent reported behaviors were eating too much or too little, binge eating, night eating and food cravings. We conclude that interprofessional approaches should target daily functioning, disrupted physical hunger cues and emotional eating to reduce disordered eating behaviors in people with SMI.

## 1. Introduction

There is a higher prevalence of eating disorders and disordered eating (a term commonly used to describe unhealthy or detrimental eating patterns that may not meet diagnostic criteria for a disorder [1]) in people with serious mental illness (SMI) including schizophrenia spectrum disorders and mood disorders (bipolar disorders and depressive disorders) [2,3].

High disordered eating rates have been reported for people with schizophrenia spectrum disorders. For example, binge eating, defined as an intensive drive to eat excessively in a short amount of time irrespective of hunger [4], has a prevalence of 4% up to 45% within this patient group [2]. Food craving, an intense desire for a specific food [5], was reported for 16% to 64% [2] of people with schizophrenia spectrum disorder. The prevalence of food addiction, an obsession with what and when to eat, and how to obtain more food when not hungry [6], ranges from 27% to 61% [2]. In addition, 4% to 30% [2] of patients reported night eating, that is characterized by evening hyperphagia and frequent awakenings accompanied by food intake, and is often co-occurring with altered circadian rhythm profiles and sleep disruptions.

Disordered eating, particularly those behaviors that lead to excess food intake, is more prevalent in people with mood disorders than in people without mental illness [7,8]. For example, 27.5% of young adults with bipolar disorder reported having either binge or emotional eating tendencies, and they were more likely to engage in stress-induced unhealthy eating behaviors [7]. In women with major depressive and/or anxiety disorders, 39% reported engaging in at least one clinically significant disordered eating behavior in their lifetime, compared to 11% in women without a depressive or anxiety disorder [8]. This might explain partly the high prevalence of obesity among people with severe mental illness.

Disordered eating is associated with a range of negative health outcomes such as: poorer nutrition status including higher intakes of energy-dense, nutrient-poor foods [9], cardiometabolic complications (higher body mass index, diabetes and metabolic syndrome) [10,11], poor mental health (depression, anxiety and psychological distress) [12,13,14] and lower quality of life [12,15]. For people with SMI, this could be an underappreciated driver of poor dietary intake and physical health disparities that lead to the 10–15-year mortality gap compared to the general population [16,17]. For instance, overweight and obesity prevalence among people with SMI is two to three times higher than in the general population [18].

Numerous factors have been implicated in the development of disordered eating in people with SMI. There is often a disruption to daily living, potentially with limited employment and supported housing [19]. Executive functioning and the food reward system can be impaired as a result of the illness making it difficult to regulate food intake [20]. This is further complicated by the disruption of usual physical hunger signals, driving disinhibition, with the onset of psychotropic medications, particularly antipsychotic medications [21]. Further symptoms of depressive illness and negative symptoms of schizophrenia can lead to hedonic and emotional eating [22]. Though, the subjective experiences with and perceptions of disordered eating of people with SMI have rarely been explored.

The majority of published qualitative literature related to nutrition in people with SMI is related to physical health ailments and associated care. Those that have a more targeted nutrition perspective tend to explore what is healthy eating and what are the barriers. For example, Barre et al. (2011) conducted 31 semi-structured interviews with people with SMI to explore the understanding of healthy eating and barriers to healthy eating [23]. Most participants described a ‘healthy diet’ in line with principles consistent with national dietary guidelines as well as internal and external barriers to healthy eating. However, there was limited focus on disordered eating behaviors, and only ‘eating for comfort’ was noted as a core internal barrier. Thus, eating for the purpose of comfort, pleasure, and filling an emotional need, that is one sort of emotional eating, makes it difficult to keep a healthy diet. [23].

We previously reported on qualitative interviews to explore the implications of dietary intake and eating behaviors for people with serious mental illness [24]. Themes emerged describing an inner conflict between food being positive for health and wellbeing (including being beneficial for body and mind, improving somatic conditions, being pleasurable, and providing an opportunity for self-efficacy) and having negative implications leading to a desire to cut out food completely (being closely related to the illness and its treatment, having negative effects on body and mind, increasing perceived stigma) [24]. The need to understand what determines and regulates eating behavior in people with serious mental illness emerged. Therefore, this study aimed to use qualitative interviews to explore the perceived determinants of eating behaviors, in particular the development, maintenance, and consequences of disordered eating patterns, in people with SMI.

## 2. Materials and Methods

### 2.1. Design

For this project, semi-structured interviews were conducted at mental health services in Ulm (Germany), Graz (Austria), and Sydney (Australia). Interviews took place between October 2019 and September 2020 and were conducted partly in person (Austria, Australia) and partly virtually (Germany, Austria) dependent on the containment measures related to the COVID-19 pandemic.

Study conduct at each site was approved by the local ethics committees: Ethics Committees of the University of Ulm (protocol code 414/19, 28 January 2020), Medical University of Graz (protocol code 32-178 ex 19/20, 20 January 2020) and South Eastern Sydney Local Health District (SESLHD) (2019/ETH12620, 14 November 2019).

### 2.2. Participants

Recruitment for the study was possible via word-of-mouth by staff, advertisements on-site, and via researchers and clinicians working in the area. Participants fulfilled the following criteria: (1) ability to provide consent, (2) older than 18 years, (3) diagnosis of a SMI (schizophrenia and related psychotic disorders, first-episode of psychosis, bipolar disorder, or major depressive disorder), and (4) current prescription of psychotropic medication. Exclusion criteria were: (1) risk of adverse mental state, distress, or discomfort as a result of participating in the interviews, (2) acute phase of mental illness, or (3) current diagnosis of an eating disorder (anorexia nervosa or bulimia nervosa). Based on previous qualitative studies in people with SMI, the goal was to recruit eight to twelve participants per site to reach anticipated thematic saturation [25].

### 2.3. Procedure

Before executing semi-structured interviews, all participants provided informed written and verbal consent. Research teams in Ulm and Sydney were able to provide financial compensation. All interviews were led by a researcher of our team (AMS, AP, ST) using a semi-structured interview guide (see Supplementary File S1) and were recorded with an audio recording device. The interview duration was set to 30 min, with a maximum of 45 min.

The interviewer started with the clarification of the intended meaning for two key terms: (1) diet referred to food choices, and (2) eating behaviors referred to aspects such as appetite, speed of eating, the structure of meals, emotional and binge eating, and overnight eating. Thereafter, the semi-structured interview guide addressed current food choices and eating behaviors and what factors are contributing to these, e.g., psychotropic medication and/or mental health state. Moreover, participants were asked to describe how mental health was impacted by food choices and eating behaviors, and whether support was offered by mental health services regarding their diet and eating behaviors.

Interviewers provided clarifications and explanations of interview questions where necessary. Participants were able to alter and/or expand their answers shortly before concluding the session.

### 2.4. Thematic Analysis

Audio recordings were transcribed verbatim. To preserve anonymity, information that could identify participants was removed and pseudonyms were used. Researchers AMS, SC and AP, who are fluent in German and fully proficient in the English language, familiarized themselves with the data and applied a thematic analysis approach, the framework method [26], by using the qualitative data analysis software MAXQDA 2020 (VERBI Software, Berlin, Germany).

The data from German interviews (Ulm) were independently coded by AMS and SC. Discrepancies in coding between the two investigators were resolved through discussion. Then, SC gathered codes that could potentially be combined into themes and debated with AMS. Based on the work in Ulm, analysis was continued similarly for data from Sydney by AMS and SC and for data from Graz by SC and AP. All three researchers (SC, AMS and AP) developed a working analytical framework and reviewed and edited the codes and emergent themes in a consensus-based approach [26]. The categories of the Palatable Eating Motives Scale (PEMS) informed the development of the working analytical framework related to themes on hedonic eating [27]. The PEMS distinguishes four motives for eating tasty food like sweets, salty snacks, fast food, or sugary drinks: coping, reward, social determinants and conformity. Finally, theme names and definitions were discussed among all authors and themes. All quotes selected to exemplify the themes were translated forward-backward if collected in German.

## 3. Results

### 3.1. Study Population

The semi-structured interviews were conducted with twelve persons in Sydney (Australia), eight persons in Ulm (Germany), and further eight persons in Graz (Austria) (see Table 1). The majority of participants (*n* = 17; 61%) were female, with age ranging from 20 to 63 years. According to the WHO body mass index criteria, 16 participants (57%) were obese with a calculated mean BMI of 31.3 kg/m^2^. Only five participants (18%) were within a healthy BMI range (20 kg/m^2^ < BMI ≤ 25 kg/m^2^).

Schizophrenia or similar psychosis-related illnesses were reported by 16 participants (57%), and 18 participants (64%) reported an affective disorder. As specified in the inclusion criteria, all participants were currently taking medication classified as psychotropics.

### 3.2. Thematic Map

**Figure 1:**
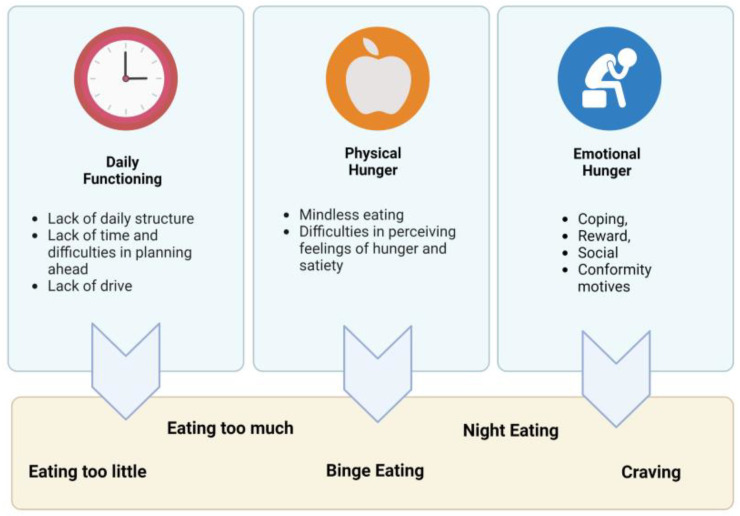
Thematic presentation of experiences with disordered eating patterns and underlying factors as reported from people with serious mental illness This figure was created with BioRender.com.

First, the participants’ subjective experiences with disordered eating behaviors are described. Second, factors are explored that contributed to the development and maintenance of these disordered behaviors according to the participants’ reports and are presented in Figure 1.

### 3.3. Subjective Experiences with Disordered Eating Behaviors

The participants rated the quality of their dietary behavior very differently, from poor to very good. However, the participants also reported that their dietary behavior has changed over time (e.g., Ulm, 01, male; Syd, 12, female), in some cases significantly, and that the quality is also subject to daily fluctuations (e.g., Ulm, 03, female; Graz, 03, female; Syd, 12, female). Participants frequently reported exhibiting unfavorable dietary behaviors in the past, i.e., eating too little or eating too much, binge eating, night eating, or craving sweets:

Some participants (e.g., Ulm, 03, female; Ulm, 04, female; Graz, 03, female; Graz, 07, female) reported that they have periods, especially during depressive episodes, when they eat little or nothing for days because of lack of drive or loss of appetite: *“Well, when it is really bad and there is also a lack of drive, then, of course, I don’t cook and then sometimes I don’t feel like making myself a sandwich so that I don’t eat anything for days”* (Ulm, 04, female). Others, however, reported that they generally eat too much, such as *“even five pieces of cake”* (Ulm 07, female).

Several participants reported binge eating, i.e., episodes of rapidly eating an excessive quantity of food linked with loss of control. One participant described: *“Then I eat this [two slices of whole wheat bread], (…) then I ate that and then I sneak there, take it all out again and eat a whole pound of bread away. Bang, boom, eaten.”* (Ulm, 07, female). As the following person explained, in extreme cases, binges can even go as far as vomiting: *“Sometimes I eat so much that I actually vomit.”* (Syd, 04, male).

Furthermore, some participants (e.g., Ulm, 01, male; Syd, 04, male; Syd, 05, female) reported night eating, i.e., difficulties sleeping, urge to eat at night, and the belief that one must eat to fall back to sleep at night: *“Yeah, and it feels like a bottomless well, and if I don’t eat at night I can’t sleep and I’m wandering the house, I feel unsettled, I, sometimes I cry because I eat, I eat when I don’t want to”* (Syd, 04, male).

Several participants (e.g., Ulm, 07, female; Syd, 05, female; Syd, 12, female) reported food cravings, i.e., an intense desire to eat a particular food, even if this is known to be rather unhealthy. It seems particularly difficult to resist these cravings during periods of stress and could even be compared to addictive cravings as well. *“When I suddenly feel like eating a specific food, I have to get it, otherwise I’ll throw a tantrum. So, yeah that’s going to be pretty hard because, (…) it’s all about discipline really. If I can discipline myself, I think I can avoid following my cravings. But if I’ve had a bad day and I’m crying and suddenly there’s this one yummy food I think will make me happy, I have to have it, no matter how unhealthy it is.”* (Syd, 12, female).

### 3.4. Subjective Factors That Contributed to the Development and Maintenance of Disordered Eating Behaviors

Participants reported several subjective determinants of disordered eating behaviors. Themes are classified as follows: (1) daily living and functional impairments, (2) physical hunger and (3) emotional hunger. The way of living and functional impairments in daily activities substantially determine the dietary behavior of the participants. Participants reported a subordinate role of physical hunger in their dietary behavior, as opposed to emotional hunger. Yet, problems with the perception of hunger and satiety were also reported. The most important driver of the dietary behavior of participants seems to be emotional hunger. Eating motives within this theme were assigned to the four categories of the PEMS: coping, reward, social, and conformity motives.

#### 3.4.1. Theme 1: Daily Functioning

As often common with SMI, participants explained how (1) a lack of daily structure, (2) a lack of time and missing planning ahead, and (3) a lack of drive impede adherence to healthy dietary behaviors.

##### Subtheme: Lack of Daily Structure

Some participants described how a lack of daily structure interfered with their dietary behavior. For instance, while one participant reported that the feeling of hunger often sets in too late and he is then overwhelmed by a ravenous appetite (Ulm, 01, male), others reported that they hardly eat any main meals due to missing day structure (Syd, 03, male; Syd, 07, male) or frequent snacking:

*“Well, yes, the problem is that, that’s why I then just probably don’t eat as many meals, healthy meals, because you eat something in between, and then you’re never hungry.”*

(Graz, 02, female)

##### Subtheme: Lack of Time and No Planning Ahead

Some participants reported not adhering to a healthy diet and not planning and preparing appropriate meals due to a lack of time or convenience. This seems to be a common theme among people struggling with mental health issues. Often, their main focus is on getting through the day and things like planning and foresight become less prioritized (e.g., Ulm, 01, male; Ulm, 06, female; Syd, 07, male; Syd, 08, male; Syd, 11, female), as outlined by one participant:

*“It’s just fast and convenient, like I’ll, I don’t organize my meals, I don’t structure it, I don’t think ahead or anything like that, I don’t cook bulk or freeze or anything like that, um, so once I get paid, I know I can buy takeaway food but until, like once the money runs out then I’m just on toast and, and cereal or whatever, um, um, so it, it’s mainly the convenience I think.”*

(Syd, 03, male)

##### Subtheme: Lack of Drive

Some participants (Ulm, 04, female; Ulm, 05, female; Ulm, 06, female; Graz, 02, female; Graz, 06, female, Syd, 06, female) reasoned that unbalanced food choices were due to lack of drive, and lack of energy, that can be associated with depression, negative symptoms of psychosis and medication side-effects, and which negatively affects shopping, cooking, and eating. This might be exacerbated by a lack of energy due to insufficient sleep because of stress and medication side effects. This can overshadow all other physical and mental needs so that the person remains completely passive in their behaviors and daily functioning tasks.

*“Erm, I eat very sporadically, actually. (…) Which has to do with the fact that I… erm, that I often lack the drive to cook or to go shopping, and if you don’t go shopping, then there’s nothing at home. (…) Then I accept it that way, and then there are also days when I don’t eat anything, so to speak. Yes. And if there is something at home, then I eat. Yes. So occasionally I eat unhealthy.”*

(Graz, 03, female)

#### 3.4.2. Theme 2: Physical Hunger

For many participants, physical hunger is less of a factor in their eating behavior. Still, (1) mindless eating and (2) difficulties in perceiving feelings of hunger and satiety were found to be challenging.

##### Subtheme: Mindless Eating

Some participants (e.g., Ulm, 05, female; Ulm, 06, female; Ulm, 08, male; Graz, 04, female; Syd, 11, female) reported that they hardly pay attention to what and why they were eating. This means that they neither consider the feeling of hunger or satiety nor do they consider the food and its taste, especially not during depressive phases. Two participants stated: *“Sometimes I feel like eating, without hunger, without knowingly acknowledging my hunger pangs.”* (Syd, 11, female) or *“Well, I don’t pay attention to my body or to the feeling. Well, I’d like to say that I could always eat.”* (Ulm, 05, female).

Another participant said this was particularly pronounced when eating in front of the television late at night: *“When I eat, I often eat in front of the television, because eating alone is just weird. So it actually runs on the side. (…) But then I usually eat [chocolate] while I’m still half asleep, and then I often don’t really notice it anymore, and then I’m always annoyed about it.”* (Ulm, 06, female).

And one woman also saw a clear connection to her obsessive–compulsive disorder. The stronger the compulsions are in a certain period of time, the less attention is paid to eating, since there is a lack of time and cognitive resources for this: *“I think the main issue is then, when I’m ready, at the table, I already think about for five other things and I don’t focus on the food and think to myself, yes, now eat quickly so that you can continue. That I don’t do it mindfully, so to say, but I’m just thinking about something else.”* (Ulm, 03, female).

##### Subtheme: Difficulties in Perceiving Feelings of Hunger and Satiety

Some participants described difficulties in perceiving feelings of hunger and satiety. Due to a loss of appetite combined with a lack of drive, participants reported eating little or no food for several days (Ulm, 04, female; Graz, 07, female). One participant reported how she got herself even into a threatening situation (*“ended up like passing out”* (Syd, 06, female) during an exercise session by misjudging her hunger pangs:

*“Appetite wise, it’s um, I’m struggling a bit in terms of like managing like my eating habits, because like I really can’t distinguish like oh am I actually hungry or it’s just my mind just trying to tell me, no you’re not hungry even though in reality I haven’t eaten anything in like the last several hours.”*

(Syd, 06, female)

In addition, an increase in appetite was particularly mentioned as a side effect of psychotropic drugs, especially olanzapine (e.g., Syd, 04, male; Syd, 12, female).

*“Olanzapine makes me so sleepy and so hungry the whole time. All I do is sleep, I could sleep from 8pm till 2pm, and as soon as I wake up I would eat then I would go back to sleep, then I would eat again, then I would go back to sleep. It was like that for a good few months thanks to the medication.”*

(Syd, 12, female)

And one participant, who is suffering from severe medication-induced weight gain, described impressively how she is unable despite all her attempts to perceive a feeling of satiety.

*“For example, I don’t have satiety, or I don’t feel full anymore [after eating enough]. I have now tried (in the hospital) to get back on track of it, but I still do not feel it. So now I sometimes feel hungry, but I don’t feel full (…). I don’t know that or I can’t perceive that, because of all the other things that distract me, like everybody is laughing at me or something (…). I can’t perceive anything, I don’t feel that and so I (…) I try then, when I’m a bit in control, try to drink a lot, that I then maybe sometime somewhere feel that inside me. I’m up to my neck somehow something like that, but basically I don’t feel that.”*

(Ulm, 07, female)

#### 3.4.3. Theme 3: Emotional Hunger

In contrast to physical hunger, other motives assigned to hedonic or emotional hunger seem to be of great importance for the participants. All four categories of eating motives included in the PEMS were discussed by participants: (1) coping, (2) reward, (3) social, and (4) conformity.

##### Subtheme: Coping (Informed by PEMS)

Some participants reported coping with negative emotions, such as depressive symptoms, anger, worry, anxiety, and frustration through emotional eating. They called it *“stress eating”* (Syd, 06, female; Ulm, 06, female), *“frustration eating”* (Graz, 02, female; Ulm, 05, female)., or *“nerve calming eating”* (Graz, 02, female) and one participant explained it in this way *“I confine to eating food to make me feel better.”* (Syd, 02, female).

One participant shared that it would help her to eat *“anything the heart desires“*, if she *“(has) trouble dealing with something and (starts) to cringe”* (Ulm, 06, female). Another participant uses it when she felt alone with her feelings and did not know how to deal with them. She reported that in this case, the emotions would provoke *“a real feeling of hunger”*. (Ulm, 04, female).

*“Yes, with specific emotions, yes, there is often a feeling of hunger or that I get an appetite for sweets or fatty things or bread or pasta. So bread and noodles are also things that I like to eat. For example, when I’m angry or worried, or afraid, and I don’t necessarily have someone to discuss these feelings with right away. So where I am simply alone with these feelings and have to see that I can deal with them. (…) And they also often actually trigger a real feeling of hunger.”*

(Ulm, 04, female)

Several participants (e.g., Graz, 02, female; Syd, 06, female) pointed out that this is rather a maladaptive strategy, as the additional food intake often ends up in a vicious circle, especially among overweight participants:

*“In fact, if one doesn’t feel good in their body, it affects your mental well-being too. (…) And that was such a loop for me. (…) I put on weight, and when one looks at oneself in the mirror, one doesn’t like themselves, one feels uncomfortable. (…) The clothes don’t fit. (…) And then one eats again in frustration. (…) One nibbles again and then it becomes more. And then the frustration becomes even greater, and that then hits the mental well-being again”*

(Graz, 02, female)

According to participants, emotional eating was also used to deal with loneliness and social conflicts. One participant explained: *“when you eat, you feel like you’re in company. If you feel a bit lonely, you eat, you feel like there’s someone extra with you, by eating satisfies up, there in your brain and your tummy, it feels relaxing.”* (Syd, 04, male). And another reported engaging in a binge session if she argues with her sister (Syd, 01, female). In contrast, there is also a participant who reacts to social conflicts with a reduced food intake up to starvation.

*“When I was fending for myself, sometimes I would go on days without eating, especially when I had a bit of a drama with my boyfriend (…). I wasn’t eating for a whole week well. Like, on good days I would eat instant noodles, on bad days I wouldn’t eat at all, and just sit on the couch and just be miserable.”*

(Syd, 12, female)

##### Subtheme: Reward (Informed by PEMS)

Seeking pleasure and reward is the driving factor in food choices for many participants. Many participants reported that the tasty foods are usually less healthy (e.g., Syd, 03, male; Syd, 04, male; Syd, 12, female), or that the healthy foods usually wouldn’t taste very good (e.g., Graz, 01, male; Syd, 04, male; Syd, 03, male; Syd, 12, female). Though, they still *“indulge”* themselves in palatable food, such as cake, despite being aware that this promotes high body weight (Ulm, 07, female). One participant argued, *“We only have this one life, let’s enjoy it. So I am a hedonist.”* (Ulm, 05, female). She further explained, *“I always eat to do myself good, because there’s not much left over that I enjoy, and the only thing that gives me a great feeling is eating.”* (Ulm, 05, female). One participant even reported that she had discovered in therapy that this emotional eating for reward had developed in her childhood, as she did not get the personal affection she needed when she was fed (Ulm, 07, female).

##### Subtheme: Social Determinants (Informed by PEMS)

Furthermore, participants raised two social factors that strongly influence dietary patterns: 1st limited financial resources, and 2nd living alone.

#### 3.4.4. Limited Financial Resources

When asked to what extent healthy eating is related to the participants’ financial situation, two contrasting attitudes or experiences were identified. One group associated healthy eating with high financial costs: *“I’d say first one has always been money, because healthy food is expensive, did you notice that salad is 10x more expensive than eating chips, for some reason.”* (Syd, 12, female), or *“It costs a lot to stick to a good food.”* (Syd, 10, female). Some of the individuals reported eating less healthy because of their economic situation: *“I always remember to eat healthy, always make sure I eat less carb, more veggies, little bit of protein. It’s just that when I don’t have the money, that’s the problem, I’ll just have to eat what I already have, like instant noodles or I’ll make some weird cooking with whatever I have and it probably wouldn’t be the healthiest option.”* (Syd, 12, female). One participant described that he always buys the cheapest food and that he does not have the possibility to pay attention to the quality and origin of the food: *“And so what I get as a pension is extremely limited and of course, how should I say, I had to orient myself to what is in my wallet. (…), there I just went in a rush: what’s available? What’s cheap? What’s for sale? I didn’t give a damn where it came from, the main thing was that I had something to eat.”* (Ulm, 01, male). Particularly noticeable is the problem for social security recipients with addiction problems, as the purchase of substances for addiction does not leave enough money for food: *“I’m just talking on my own experience, and I know people they go just with being on Centrelink (social security service), you don’t have enough money to, to, like feed yourself for two weeks if you’ve got bills and especially if you’ve got bills and (…) you smoke, so a lot of that goes to buying cigarettes and you don’t have enough, you don’t leave enough money for you to sort of eat without you know, if you put, if you like to drink alcohol you’ll buy that before you’ll buy food, if you like cigarettes you’ll buy that before you’ll buy food (…).”* (Syd, 03, male).

In contrast to this group, other participants shared the attitude that healthy food is not inevitably expensive and that, with good financial management, a healthy diet can be achieved despite a low income:

*“Because I don’t buy so (…) so elaborately, and so expensively. (…) Basically, I have never done that. (…) But I still make sure that it is healthy. (…) In that regard, I don’t save money or use only cheap products, that’s not the case. (…) Then I eat less frequently something delicious, but healthy. (…) So I do pay attention to that.”*

(Graz, 02, female)

*“I can’t eat out as much as I’d like to at expensive restaurants. But if you eat wisely and manage, manage your budget, you can eat sensibly as well. If it, obviously I can’t have fish every night, um, the more exotic foods I can’t have all the time. But they, those main food groups I think I’m pretty well, most of the time.”*

(Syd, 08, male)

#### 3.4.5. Living Alone

Participants living alone commented that it is *“always a bit dumb”* (Ulm, 06, female) to go shopping, cook and eat alone. Poor self-esteem plays a significant role in this context (Graz, 03, female; Ulm, 06, female).

*“I have to cook for myself alone and there is often just a lack of this: am I even worth it to do this for myself now, so is it even that important to cook something great now or is it enough if I somehow just put a loaf of bread in or a pizza or something like that?”*

(Ulm, 06, female)

In addition, there is the lack of drive mentioned at the beginning and the difficulty of cooking small volumes, which results in the participants having to eat the same food for several days (Graz, 02, female; Graz, 03, female). However, two participants have now found a way to deal with these challenges by deliberately initiating joint meals with family and friends. This enabled them to cook proper meals again (Syd, 08, male), and food also made them *“happy and cheerful again”* (Ulm, 01, male).

##### Subtheme: Conformity (Informed by PEMS)

The conformity motive involves food intake in response to external pressure, e.g., adapting food intake to family behavior to fit in, or changing food intake because family and friends want us to. Conformity motives often seem to have a rather negative impact on the dietary behavior of those affected, as individual goals and needs are subordinated due to other factors, which in turn may lead to a positive energy balance that promotes weight gain.

Some participants described how their dietary behavior is shaped by the behaviors of family members. For example, the meal selection and the food supply are influenced by the preferences of the other family members, which means that sometimes very fatty meals are served. *“So I eat what the others eat, too”* (Ulm, 05, female). 

One participant also described that the mother would buy sweets, which then leads to a desire for sweets. *“I think the barriers are that I’m always tempted to buy those chocolates and junk food and mum buys them a lot as well, then tells me that she’s not going to buy them anymore but continues to buy them and when they’re in the cupboard I have more temptation to eat them than when they’re not there.”* (Syd, 02, female).

The amount of food intake is also strongly related to the family members’ behaviors. Indeed, one participant described his mother as *“a big portion giver”* (Syd, 04, male). One woman described how her husband’s offerings of food make it difficult for her to eat only the amount she had intended: *“He (husband) wants to do something good for his wife and always offers me something, if he eats something. Would you like something too? And I said no once, and he said, oh come on, I have (…) if you want something. Then I said no again, but at some point I can’t anymore and then it’s over and then I’ve lost. That’s lost.”* (Ulm, 07, female).

However, for one participant who was seriously underweight at some point, conformity motives could also have a positive effect on dietary behavior and body weight. This participant reported that the significant weight loss only began when she moved out of her parents’ home and sought to live independently. Now, in turn, it was an advantage that she is no longer living alone, but can feed her partner and herself at the same time. In contrast, the direct pressure to eat more from her previous partner was perceived as negative by the participant.

*“Because I think it was easy to, when I was living at mum’s house, it was easy to because food was always on the table. So, I don’t know how, if that’s a good or positive thing but it was very easy to put on weight at mum’s house. And it was very easy to just not eat when you start living by yourself and have to prepare your own meals.”*

(Syd, 12, female)

*“Thank god I live with my partner and I have someone to feed. So, I’m feeding him, I’m feeding me.”*

(Syd, 12, female)

*“First time I ever got fat was with my previous boyfriend ages ago, he always force feeds me, and forces me to finish my meal. And he would serve me a meal for three people and he would tell me to finish it all. And it was like that for a whole year.”*

(Syd, 12, female)

## 4. Discussion

In this study, we aimed to explore determinants of disordered eating by conducting 28 semi-structured interviews with people with SMI in three different countries. We derived three main themes connected to disordered eating patterns: daily functioning, physical hunger and emotional hunger. In the category of daily functioning, people with SMI mentioned problems with a lack of daily structure, lack of time, missing planning ahead as well as a lack of drive. Besides problems in daily functioning, disruption of physical hunger cues (mindless eating, difficulties in perceiving feelings of hunger and satiety) and emotional hunger (coping, reward, social and conformity factors) seem to be important causes for the development of disordered eating behaviors such as eating too little or too much, binge eating, night eating and craving.

People with SMI frequently withdraw socially, discontinue employment and/or education, stop doing everyday tasks and stop pursuing their hobbies [19]. As a result, social contacts and a regular daily structure are disrupted. Daily functioning and daily structure are especially impaired during acute mental crises. These functional challenges impose social, occupational, and financial, but also health-related burdens on people with SMI [28]. Lack of drive, one of the major symptoms of people with SMI, creates a lack of incentive to prepare meals and apply adequate self-care, which constituted a very important theme in our interviews as well. Time scarcity and lack of meal planning are known to lead to unhealthy lifestyles, i.e., the consumption of readymade, ultra-processed meals [29]. Hence, we deduce that special support in daily activities and daily structure should be provided, e.g., by social workers and nurses to improve daily functioning and the dietary behavior of people with SMI [30]. In addition, interventions based on the principles of motivational interviewing could help in promoting health behaviors and behavioral change [31]. Though no superiority of a motivational interviewing-based intervention compared to routine care could be demonstrated in a quasi-experimental pilot study in people with SMI in sheltered housing, qualitative interviews underline the acceptability of, and satisfaction with, the intervention among mental health workers. The professionals positively emphasized that the intervention raised the awareness of the service users and that initial, albeit small, behavioral changes were achieved [32].

Our participants reported difficulties in perceiving feelings of hunger and satiety. It is well known that the regular intake of psychotropic medications interferes with satiety mechanisms, both decreasing and increasing appetite [33]. Moreover, imbalances of satiety hormones ghrelin and leptin have been reported in people with SMI [34,35]. SMIs are further connected to periods of chronic stress. Chronic stressors along with lack of sleep lead to disrupted cortisol responses and result in weight gain, with ghrelin playing a role in increased food cravings and reward-driven eating behaviors [36]. Targeted mindful eating interventions could help with raising awareness of the food consumed and improve the perception of hunger and satiety in people with SMI. Mindful eating has been associated with less impulsive eating, reduced calorie consumption and healthier snack choices [37]. In a pre–post pilot study, a mindful eating program focusing on self-efficacy and self-regulation was found to be helpful to address unhealthy eating among 46 participants with SMI [38].

From our interviews, we deduced that emotional eating is a much more meaningful factor in food intake than physical hunger. Tuncer et al. demonstrated that the prevalence of emotional eating behavior is 49% among people with SMI [39]. An increasing number of prospective studies have shown that emotional eating predicts weight gain in adults [40], therefore emotional eating may be one behavioral mechanism that connects depression to the development of obesity. For our participants, especially the PEMS sub-domains of coping and reward have shown to be of special importance. Using food as a coping strategy is connected to perceived stress, emotion-triggered eating, binge eating and greater BMI. Eating as a reward was found to be connected to anxiety, depression, and greater BMI in females, and more anger/frustration in males [27]. This might partly explain why people with SMI are primarily referring to negative implications, such as guilt and stigma, when talking about their dietary behavior [24]. Emotional hunger may be avoided by early identification and training in other means of coping with stress and stress reduction in mental health services (e.g., behavioral therapy, skills training [41], or mindfulness-based stress reduction (MBSR) [42]. Moreover, initiation of special living and eating conditions (eating in company, cooking workshops) could assist people with SMI to address social and conformity factors of their dietary behavior.

**Figure 2:**
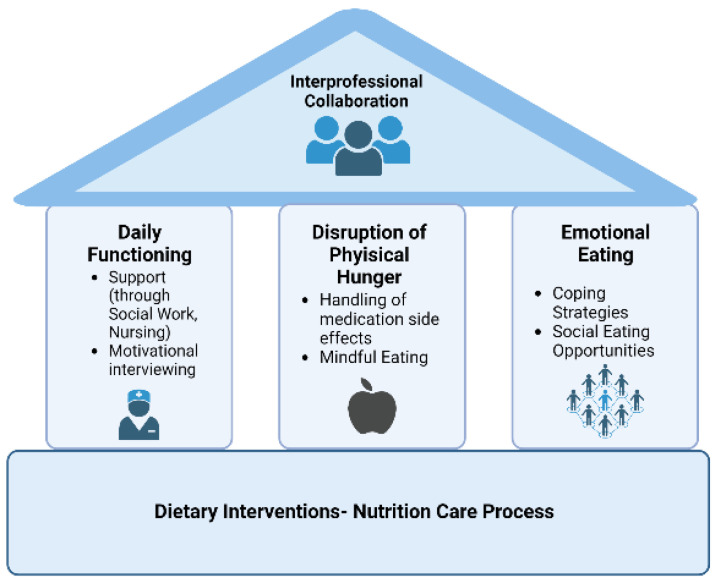
Future interventions to improve dietary behavior focused on interprofessional collaboration. Figure created with BioRender.com.

Given the detrimental effects of disordered eating on physical and mental health, dietary behavior should also be addressed as part of mental health care in an interprofessional approach. Formal screening processes need to be employed that trigger referral to appropriate clinicians. Figure 2 illustrates a model for collaborative care of people with SMI and disordered eating. Consistent across the three pillars is the Nutrition Care Process as a foundation treatment, facilitated by dietitians. This can be complimented, for example, by social work and occupational therapists for daily functioning challenges, physicians and nurses for disruption to physical hunger, and psychologists and psychotherapists for emotional eating.

Most of these are also addressed in the recently published clinical guidelines for the use of lifestyle-based mental health care in major depressive disorder. The need for interprofessional collaborations and networks in a biopsychosocial-cultural framework is highlighted as key implementation considerations [43]. However, in our interviews diet-related support (e.g., services listed as pillars in Figure 2) was reported not to be widely available in mental health settings [24]. One reason therefore might be that mental health professionals report poor literacy in nutritional medicine [44]. For this purpose, training for health professionals in the recognition and treatment of disordered eating behaviors in people with SMI must be established. Awareness of possible medication side effects regarding appetite and body weight, as well as of emotional eating in people with SMI must be built.

Our study has several strengths: This is one of the first studies describing the subjective determinants of disordered eating in people with SMI. Our study was conducted in three different countries; at the site in Australia, nutritional interventions are offered as part of routine care, at the sites in Austria and Germany, nutrition is not primarily part of mental health care. A cross-country comparison would be of interest, as determinants of dietary behavior might differ based on received treatments and based on cultural specificities. However, this was beyond the scope of this study. Given the nature of qualitative studies and small sample size, our results may not be representative. Moreover, people with a higher interest in diet and diet-related support or more pronounced diet-related problems may be more likely to take part in this kind of research. Most of our study participants were female, however, a gender-specific analysis was not possible within this study’s scope.

## 5. Conclusions

Disordered eating behaviors are common in people with SMI and are overlooked by mental health services. Interprofessional approaches targeting daily functioning, disrupted physical hunger cues and emotional eating are urgently needed to reduce disordered eating behaviors in people with SMI.

## Figures and Tables

**Table 1:** Description of the study population (m = mean, sd = standard devaiation).

	Australia (*n* = 12)	Austria (*n* = 8)	Germany (*n* = 8)	Total (*n* = 28)
sex, female; *n* (%)	7 (58%)	5 (63%)	5 (63%)	17 (61%)
age, in years; m (sd)	40.0 (15.6)	43.1 (11.1)	48.6 (10.7)	43.3 (13.5)
schizophrenia or related disorders (ICD-10 F2); *n* (%)	11 (92%)	2 (25%)	3 (38%)	16 (57%)
affective disorders (ICD-10 F3); *n* (%)	5 (42%)	6 (75%)	5 (63%)	18 (64%)
body-mass-index (BMI), in kg/m^2^; m (sd)	31.3 (5.0)	26.8 (3.7)	35.6 (7.3)	31.3 (6.4)
obesity (BMI > 30 kg/m^2^); *n* (%)	7 (58%)	2 (25%)	7 (88%)	16 (57%)

## Data Availability

Data are only accessible upon reasonable request because of data security issues.

## Supplementary Materials

The following supporting information can be downloaded at: https://www.mdpi.com/article/10.3390/ijerph20010442/s1, File S1: Guide for service user interviews.
